# Constraints on Energy Intake in Fish: The Link between Diet Composition, Energy Metabolism, and Energy Intake in Rainbow Trout

**DOI:** 10.1371/journal.pone.0034743

**Published:** 2012-04-09

**Authors:** Subramanian Saravanan, Johan W. Schrama, A. Claudia Figueiredo-Silva, Sadasivam J. Kaushik, Johan A. J. Verreth, Inge Geurden

**Affiliations:** 1 Aquaculture and Fisheries Group, Wageningen Institute of Animal Sciences (WIAS), Wageningen University, Wageningen, The Netherlands; 2 Institut National de la Recherche Agronomique (INRA), UR1067, Nutrition, Metabolism and Aquaculture (NuMeA), Pôle d'Hydrobiologie INRA, Saint Pée-sur-Nivelle, France; Institut Pluridisciplinaire Hubert Curien, France

## Abstract

The hypothesis was tested that fish fed to satiation with iso-energetic diets differing in macronutrient composition will have different digestible energy intakes (DEI) but similar total heat production. Four iso-energetic diets (2×2 factorial design) were formulated having a contrast in i) the ratio of protein to energy (P/E): high (H_P/E_) vs. low (L_P/E_) and ii) the type of non-protein energy (NPE) source: fat vs. carbohydrate which were iso-energetically exchanged. Triplicate groups (35 fish/tank) of rainbow trout were hand-fed each diet twice daily to satiation for 6 weeks under non-limiting water oxygen conditions. Feed intake (FI), DEI (kJ kg^−0.8^ d^−1^) and growth (g kg^−0.8^ d^−1^) of trout were affected by the interaction between P/E ratio and NPE source of the diet (P<0.05). Regardless of dietary P/E ratio, the inclusion of carbohydrate compared to fat as main NPE source reduced DEI and growth of trout by ∼20%. The diet-induced differences in FI and DEI show that trout did not compensate for the dietary differences in digestible energy or digestible protein contents. Further, changes in body fat store and plasma glucose did not seem to exert a homeostatic feedback control on DEI. Independent of the diet composition, heat production of trout did not differ (P>0.05). Our data suggest that the control of DEI in trout might be a function of heat production, which in turn might reflect a physiological limit related with oxidative metabolism.

## Introduction

Fish under farming conditions are mostly fed pre-set amounts of a single feed type so that the fish cannot compensate feed intake (FI) for the eventual lack of a particular nutrient or for energy content, which may lead to reduced growth. Thus, predicting the feed ration close to the voluntary FI level of fish as a function of diet composition and culture conditions is essential to maximize growth rate and feed use and also to minimize feed wastage in the aquatic environment. This requires a better understanding of the dietary, physiological and environmental factors affecting FI and their underlying mechanisms.

Compared to mammals, mechanisms controlling FI are relatively less explored in fish. It was stated that “fish like other animals, eat to satisfy their energy requirements” [1]. Indeed, among the dietary factors, the digestible energy (DE) content has been widely suggested to be a major determinant of FI control in several fish species such as rainbow trout, *Oncorhynchus mykiss* [2], [3], [4], [5], Atlantic salmon, *Salmo salar* [6], Atlantic cod, *Gadus morhua* [7], European seabass, *Dicentrarchus labrax* [8], turbot, *Scophthalmus maximus* [9] and Channel catfish, *Ictalurus punctatus* [10].

In contrast, some studies have shown that fish do not regulate their FI based on dietary DE density as a whole, as seen in rainbow trout [11], [12], Atlantic salmon [13], Arctic charr, *Salvelinus alpinus* [14] and European seabass [15], suggesting a possible role of energy or nutrient utilization and thus of DE source in FI regulation in fish. Recently, Tran Duy et al. [16] studied the effect of changes in DE source (fat vs. starch) on FI in Nile tilapia, *Oreochromis niloticus* and found similar dry matter FI but different digestible energy intake (DEI) as affected by the DE source of the diet. One striking observation in that study was the similar total heat production of fish, irrespective of the diet-induced differences in ingested (DE) and retained (RE) energy. Based on the observation of similar heat production, calculated as the difference between metabolisable and retained energy, the authors postulated the involvement of heat production in the control of FI in Nile tilapia. Therefore, the present study further investigates the relation between heat production and the effect of macronutrient composition on FI and DEI in another teleost model, rainbow trout. We hypothesized that rainbow trout fed to satiation with iso-energetic diets, differing in protein to energy ratio (P/E) as well as in non-protein energy (NPE) source, would result in different DEI but with similar heat production.

## Materials and Methods

The experiments were conducted following the Guidelines of the National Legislation on Animal Care of the French Ministry of Research (Decree 2001-464 of May 29, 2001) and were approved by the Ethics Committee of INRA (according to INRA 2002-36 of April 14, 2002).

### Diets

Four diets were formulated in a 2×2 factorial design with protein to energy ratio (P/E) and non-protein energy (NPE) source as main factors, each consisting of two levels, being ‘high’ vs. ‘low’ and ‘fat’ vs. ‘carbohydrate’, respectively. The formulation and ingredient composition of diets are shown in Table 1. In order to have identical nutrient and energy density between diets, 15% of cellulose was included in the fat diets. We thus had four diets (Table 1) *viz.*, high P/E ratio with fat as energy source (H_P/E_F), high P/E ratio with carbohydrate as energy source (H_P/E_C), low P/E ratio with fat as energy source (L_P/E_F) and low P/E ratio with carbohydrate as energy source (L_P/E_C). As expected, all four diets resulted in similar digestible energy content (∼18 kJ g^−1^) and contrast in P/E ratio between H_P/E_ diets (∼26 mg kJ^−1^) and L_P/E_ diets (∼14 mg kJ^−1^). The ingredient mixtures of each diet were extruded through a 2 mm die, dried, sieved, and stored in plastic bags (feed extrusion plant, INRA Donzacq, France). The analyzed nutrient compositions of the four diets are detailed in Table 1.

**Table 1:** Formulation, ingredient composition and analyzed nutrient content of experimental diets.

	Diets^1^			
	H_P/E_F	H_P/E_C	L_P/E_F	L_P/E_C
*Ingredients (%)*				
Protein mixture^2^	66.0	66.0	35.9	35.9
Oils^3^	11.0	1.0	19.1	9.1
Gelatinized maize starch^4^	5.0	30.0	24.3	49.3
Cellulose^5^	15.0	0.0	15.0	0.0
Other^6^	3.0	3.0	5.7	5.7
*Analyzed nutrient content on DM basis (g kg^−1^)*				
Dry matter (DM; g kg^−1^ diet)	938	924	949	947
Crude protein (N×6.25)	519	511	276	261
Crude fat	152	34	207	143
Total carbohydrates^7^	254	380	444	528
Starch	49	303	246	456
Ash	75	75	73	68
Gross energy (GE; kJ g^−1^)	22.8	20.6	22.8	21.2
Digestible energy (DE; kJ g^−1^)	18.70	18.27	18.74	18.19
DP/DE (mg kJ^−1^)^8^	26.5	26.8	14.1	13.7

1 H_P/E_F - High P/E ratio diet with fat as main non-protein energy source; H_P/E_C - High P/E ratio diet with carbohydrate as main non-protein energy source; L_P/E_F - Low P/E ratio diet with fat as main non-protein energy source; L_P/E_C - Low P/E ratio diet with carbohydrate as main non-protein energy source.

2 Protein mixture (% mixture): 50% fishmeal (Sopropêche 56100 Lorient, France), 16.5% soybean protein concentrate (Sopropêche 56100 Lorient, France), 16.5% pea protein concentrate (Roquette 62080 Lestrem, France), 16.5% wheat gluten (Roquette 62080 Lestrem, France) and 0.5% DL methionine (Ajinomoto Eurolysine 75017 Paris, France).

3 Oils: rapeseed oil (Daudruy 59640 Dunkerque, France) in H_P/E_ diets; 5% (% diet) fish oil (Sopropêche 56100 Lorient, France) and the remaining part from rapeseed oil in L_P/E_ diets.

4 Gelatinized maize starch: Roquette 62080 Lestrem, France.

5 Cellulose: Rettenmeier et Sohne 73494 Rosenberg, Germany.

6 Other (% diet): 2% Diamol (indigestible marker, Diamol GM, Franz Bertram Hamburg, Germany); 1% vitamin and mineral premix (INRA UPAE 78200 Jouy en Josas). For L_P/E_-diets 0.4% CaCO_3_, 1.8% Ca(HPO_4_)_2_, and 0.5% Na_2_CO_3_ were added.

7 Calculated as, total carbohydrates (starch, free sugars, cellulose) = 1000−(crude protein+crude fat+ash).

8 DP/DE (Digestible protein to digestible energy ratio) = (Crude protein×% apparent digestibility coefficient of crude protein)/(gross energy×% apparent digestibility coefficient of gross energy- see table 4).

### Feeding trial and sampling

Rainbow trout (*O. mykiss*) were obtained from the same parental stock (INRA Lées-Athas fish farm, France) and were transferred to the experimental facilities of INRA (Donzacq, France) where they were acclimatized to the rearing conditions prior to the start of the feeding trial. The experimental setup consisted of 12 independent circular tanks (150 L) in a flow-through system (flow rate, 0.4 L sec^−1^; water renewal in tank minimum 8 times per h) supplied with natural spring water having a temperature of 16±1°C (mean ± SD), average pH (7.4), ammonia (<0.05 mg L^−1^), nitrite (<0.02 mg L^−1^), nitrate (<15 mg L^−1^), dissolved oxygen (DO; >8.5 and >7.0 mg L^−1^ respectively in inlet and outlet) under natural light regimen (February-April). At the start of experiment, fish (32.4 g initial body weight) were sorted for homogenous size and randomly allotted among the 12 tanks (35 fish/tank). Diets were assigned randomly to triplicate tanks and hand-fed twice daily to visual satiation (i.e., feed distributed until the fish stop displaying active feeding) in morning and afternoon. In total, the feeding trial lasted for 7 weeks, during the first 6 weeks (growth period) we assessed feed intake, growth and nutrient utilisation, and then fish were allowed to recover for 1 week (recovery period) before post-prandial sampling. During the growth period, mortality was monitored daily and fish were group weighed every 2 weeks to calculate intermediate growth and feed intake. A random sample of 36 h feed deprived fish were euthanized (overdose of anaesthesia, 2-phenoxy-ethanol) and stored at −20°C for subsequent analyses of whole body composition, at the beginning (35 fish) and end (8 fish/tank) of the growth period. At the end of the 6 weeks, all fish were counted and weighed to calculate the final body weight of fish. The fish were then continued to be fed their respective diets for a period of 1 week (recovery period) prior to post-prandial blood sampling. At 7 h post-feeding, nine fish per dietary treatment were sampled for blood. The blood was drawn from the caudal vein and transferred into a vial containing 20 µl anticoagulant (2 g potassium oxalate+1 g sodium fluoride in 100 ml distilled water). Blood samples were centrifuged (3000 G, 10 min) and the plasma obtained were stored at −20°C until analyses of glucose and triglycerides.

### Digestibility study

In parallel to the 6-week feeding trial, a separate 4-week digestibility trial was conducted at the INRA fish rearing unit (St Pée-sur-Nivelle, France) with rainbow trout from the same stock as in the feed intake study. Fifteen fish (mean body weight, 65 g) were stocked in 12 cylindro-conical tanks (60 L) connected to an automatic faeces collection unit [17], the diets were assigned randomly among tanks in triplicates. The tanks received continuous supply of water (14±1°C; mean ± SD) from the recirculation water system and were maintained at uniform conditions throughout the experiment. Prior to faeces collection, fish were acclimatized for a week to the experimental conditions and to their respective experimental diets. Diamol (acid insoluble ash, AIA) was added into the feed as inert marker for determining digestibility. Fish were fed twice daily (1.5% of body weight) and faeces collected twice daily over 3 weeks, pooled per tank and stored at −20°C.

### Chemical analyses

Whole fish from each tank were ground, pooled and fresh moisture content was determined. Fish and faeces were subsequently freeze-dried before further analyses. The nutrient compositions of fish, diet and faeces were analyzed according to the following procedures. Feed, faeces and whole body samples were analyzed for dry matter (105°C for 24 h), protein (Kjeldahl; N×6.25) after acid digestion, fat content of feed and faeces [18] using dichloromethane instead of chloroform and the fat content of fish by petroleum ether extraction (Soxhlet; 40–60°C) and gross energy content by adiabatic bomb calorimeter (IKA-Werke C5000). Ash contents were determined by combustion in muffle furnace (550°C for 12 h). The same ash samples of feed and faeces were used to determine acid insoluble ash [19]. Starch content was determined as glucose, using the amyloglucosidase/hexokinase/glucose-6-phosphate dehydrogenase method after ethanol (40%) extraction and starch decomposition in dimethylsulfoxide/HCl [20]. Plasma glucose and triglycerides were determined following the procedures provided in the commercial kits, Glucose RTU (n° 61269) and Triglycérides (PAP 150 n° 61236) from Bio-Mérieux, Marcy-L'Etoile, France.

### Calculations

The mean individual initial (W_i_) and final (W_f_) body weight of fish was obtained dividing the total initial and final fish biomass of the tank by the number of fish present in tank at start and end of study respectively. Absolute growth of fish (in g d^−1^) was calculated as the difference between mean individual final (W_f_) and initial (W_i_) body weight of fish per tank divided by duration of experimental period (t). The geometric mean body weight (W_G_; in g) is calculated as 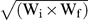, from which mean metabolic body weight (MBW_G_; in kg^0.8^) was calculated as (W_G_/1000)^0.8^. Growth rate on metabolic body weight (GR_MBW_; in g kg^−0.8^ d^−1^) was calculated as (W_f_−W_i_)/(MBW_G_×t). Daily growth coefficient (DGC, in % d^−1^) was calculated as 100×(W_f_^1/3^−W_i_^1/3^)/t.

Absolute feed intake (FI_ABS_; g DM fish^−1^ d^−1^) was calculated on dry matter (DM) basis as FI_tot_/(n×t) where FI_tot_ is the total feed intake per tank (in g DM) over experimental period, n is the number of fish in tank and t is the experimental period. FI as fed (g fish^−1^ d^−1^) was calculated in similar way as FI_ABS_ but on as fed basis. Feed intake of fish expressed as a percentage of body weight (FI_PCT_; % d^−1^) was calculated as (FI_ABS_/W_G_)×100/t and feed intake per metabolic body weight (FI_MBW_; g DM kg^−0.8^ d^−1^) was calculated as FI_ABS_/MBW_G_. Feed gain ratio (FGR; dry matter intake/wet weight gain) was calculated on DM basis as FI_MBW_/GR_MBW_.

Apparent digestibility coefficients (ADC, in %) of dry matter, crude protein, crude fat, total carbohydrate, gross energy and ash were calculated for each tank using acid insoluble ash (AIA) as inert marker as described previously [16]. Apparent digestibility coefficients were calculated as ADC_X_ = (1−(AIA_diet_/AIA_faeces_)×(X_faeces_/X_diet_))×100, where X represents dry matter, crude protein, crude fat, total carbohydrate, gross energy and ash, AIA_diet_ and AIA_faeces_ are the AIA content in the diet and faeces, respectively and X_diet_ and X_faeces_ are the quantity of X in the diet and faeces, respectively.

The parameters of nitrogen balance (mg N kg^−0.8^ d^−1^) and energy balance (kJ kg^−0.8^ d^−1^) were calculated per tank, without changes as described earlier [16]. The gross nitrogen intake (GNI) was calculated as product of total feed intake (g DM kg^−0.8^ d^−1^) and nitrogen content of feed (mg g^−1^). The digestible nitrogen intake (DNI) was calculated as product of GNI and ADC of nitrogen (%). Faecal nitrogen loss (FN) was calculated as the difference between GNI and DNI. The retained nitrogen (RN) was calculated as the difference between nitrogen content of final and initial fish carcass. Branchial and urinary nitrogen loss (BUN) was calculated as difference between DNI and RN. Parameters of energy balance were calculated as follows: gross energy intake (GEI) as the product of feed intake (g DM kg^−0.8^ d^−1^) and energy content of the diet; digestible energy intake (DEI) as product of GEI and ADC of energy; metabolisable energy intake (MEI) was calculated as the difference between DEI and the branchial and urinary energy loss (BUE), which was estimated as BUE = (BUN×24.85)/1000, where 24.85 is the amount of energy (in kJ) equivalent to 1 g excreted nitrogen, assuming that all nitrogen is excreted as NH_3_–N [21]; retained energy (RE) as the difference between energy content of final and initial fish carcass. The total heat production (H) was calculated as the difference between metabolisable energy intake (MEI) and retained (RE) energy from the energy balance. Similarly, the fat balance (mg kg^−0.8^ d^−1^) was calculated per tank. The gross fat intake (GFI) was calculated as product of total feed intake (g kg^−0.8^ d^−1^) and fat content of feed (mg g^−1^). The digestible fat intake (DFI) was calculated as product of GFI and ADC of fat (%). Faecal fat loss (FF) was calculated as the difference between GFI and DFI. The retained fat (RF) was calculated as difference between fat content of final and initial fish carcass.

### Statistical procedure

Statistical analyses were performed using SAS 9.2 (SAS Institute, Cary, NC, USA). Data were analyzed for the effect of P/E ratio, type of NPE source and their interaction by two-way ANOVA (PROC GLM). Normal distribution of the residuals was verified using Kolmogorov-Smirnov's test (PROC UNIVARIATE). The faecal fat loss (FF) overruled the assumption of normal distribution (P<0.05) and logarithmic data transformation satisfied the assumptions. In the case of a significant interaction, post-hoc pair wise comparison of means was done using Tukey-Karmer test.

## Results

### Feed intake and growth

Feed intake (in g fish^−1^ d^−1^, g DM fish^−1^ d^−1^, % d^−1^, and g DM kg^−0.8^ d^−1^), growth (in g d^−1^ and g kg^−0.8^ d^−1^), and feed gain ratio (FGR) were significantly affected by the P/E ratio and by the NPE source of the diet with a highly significant interaction between both factors (Table 2).

**Table 2:** Voluntary feed intake and growth performance of rainbow trout fed the experimental diets for 6 weeks^1^.

	Diets^2^					*P*- value		
	H_P/E_F	H_P/E_C	L_P/E_F	L_P/E_C	Pooled SEM	P/E ratio	NPE source	P/E×NPE
Growth period (d)	42	42	42	42	-	-	-	-
No. of tanks	3	3	3	3	-	-	-	-
No. of fish/tank	35	35	35	35	-	-	-	-
Survival (%)	98.1	98.1	96.2	89.5	1.90	0.025	0.118	0.118
Initial body weight (g)	32.4	32.5	32.3	32.4	0.35	0.792	0.792	1.000
Final body weight (g)	103.7^a^	96.6^ab^	84.4^b^	59.5^c^	3.26	<0.001	0.001	0.025
*Feed intake (FI)*								
FI as fed (g fish^−1^ d^−1^)	1.82^a^	1.59^b^	1.53^b^	1.00^c^	0.044	<0.001	<0.001	0.010
FI_PCT_ (% d^−1^)	2.9^a^	2.6^b^	2.8^ab^	2.2^c^	0.05	<0.001	<0.001	0.018
FI_ABS_ (g DM fish^−1^ d^−1^)	1.70^a^	1.46^b^	1.45^b^	0.95^c^	0.042	<0.001	<0.001	0.013
FI_MBW_ (g DM kg ^−0.8^ d^−1^)	16.6^a^	14.7^b^	15.4^ab^	11.6^c^	0.29	<0.001	<0.001	0.012
*Growth*								
Absolute (g d^−1^)	1.70^a^	1.53^ab^	1.24^b^	0.65^c^	0.078	<0.001	0.001	0.026
GR_MBW_ (g kg^−0.8^ d^−1^)	16.5^a^	15.3^ab^	13.2^b^	7.9^c^	0.60	<0.001	<0.001	0.009
DGC	3.6^a^	3.3^ab^	2.9^b^	1.8^c^	0.13	<0.001	<0.001	0.008
FGR (DM intake/wt.gain)	1.01^a^	0.96^a^	1.17^b^	1.48^c^	0.037	<0.001	0.008	0.001

DM, dry matter; FI_PCT_, Feed intake per percentage body weight; FI_ABS_, Absolute feed intake; FI_MBW_, Feed intake per metabolic body weight; DGC, Daily growth coefficient; FGR, Feed gain ratio.

1 Values represent least squares (LS) means (n = 3), row means with different superscript letters were significantly different and assigned only if interaction effect was significant (P<0.05).

2 H_P/E_F - High P/E ratio diet with fat as main non-protein energy source; H_P/E_C - High P/E ratio diet with carbohydrate as main non-protein energy source; L_P/E_F - Low P/E ratio diet with fat as main non-protein energy source; L_P/E_C - Low P/E ratio diet with carbohydrate as main non-protein energy source.

Within H_P/E_ and L_P/E_ groups, feed intakes were affected by the type of NPE, being lower in trout fed carbohydrate relative to fat as NPE source. The effect of NPE source on FI was greater with L_P/E_ diets (∼20% difference) than with H_P/E_ diets (∼11% difference); with lowest intakes registered in trout fed the L_P/E_C diet (11.6 g DM kg^−0.8^ d^−1^). Trout fed the diets containing fat as NPE source, i.e. H_P/E_F (16.6 g DM kg^−0.8^ d^−1^) and L_P/E_F (15.4 g DM kg^−0.8^ d^−1^) had similar dry matter intakes, irrespective of P/E ratio. Intakes of trout fed the diets with carbohydrate as NPE source were lower at low than at high P/E ratio. At high P/E ratio, growth (g kg^−0.8^ d^−1^) was not significantly different between groups fed diet H_P/E_F and H_P/E_C, despite their different feed intakes. At low P/E intake, growth was lower in trout fed carbohydrate (L_P/E_C) relative to fat (L_P/E_F) as NPE source. The lowest growth was found in fish fed diet L_P/E_C (7.9 g kg^−0.8^ d^−1^), being 1.6 times lower than that of the L_P/E_F group. Remarkably, growth of trout fed the L_P/E_F diet (with only DP/DE of 14 mg kJ^−1^) did not differ significantly from that of fish fed diet H_P/E_C (with DP/DE of 26 mg kJ^−1^). The FGR was also affected by a significant interaction between both factors (NPE source and P/E ratio), being higher in trout fed carbohydrate compared to fat at the low P/E ratio, but not at the high P/E ratio at which FGR was not affected by the NPE source.

### Body composition

The initial and final body compositions of the trout are shown in Table 3. Except for dry matter, other parameters (protein, fat, ash, and energy) of final body composition were affected (P<0.01) by P/E ratio of diet. Similarly, NPE source of diet affected (P<0.01) all parameters except protein and ash. There was no significant interaction between both effects on final body composition, except for ash content. Whole body protein content of fish fed L_P/E_ diets was about 11% lower than in those fed with H_P/E_ diets (P<0.001). Compared to initial body protein content, fish fed with L_P/E_ diets had 7.5% lower protein content. Final body fat content increased in all groups compared to initial body fat content. Whole body fat content was 24% significantly higher in trout fed with L_P/E_ diets (low P/E ratio) and 44% higher in groups fed diets containing fat as NPE source (P<0.01).

**Table 3:** Effect of dietary treatments on final body composition (on fresh weight basis) of rainbow trout fed the experimental diets for 6 weeks^1^.

		Final body composition							
*Unit in g kg^−1^*	Initial bodycomposition	Diets^2^					*P*- value		
		H_P/E_F	H_P/E_C	L_P/E_F	L_P/E_C	Pooled SEM	P/E ratio	NPE source	P/E×NPE
Dry matter (DM)	220	278	251	285	257	4.1	0.125	<0.001	0.871
Protein	153	156	162	143	140	3.4	<0.001	0.632	0.263
Fat	34	94	61	111	81	3.9	0.001	<0.001	0.684
Ash	26	21^a^	21^a^	19^b^	20^ab^	0.5	0.008	0.223	0.042
Energy (kJ g^−1^)	5.0	7.5	6.3	8.1	6.8	0.16	0.015	<0.001	0.913

1 Values represent least squares (LS) means (n = 3), row means with different superscript letters were significantly different and assigned only if interaction effect was significant (P<0·05).

2 H_P/E_F - High P/E ratio diet with fat as main non-protein energy source; H_P/E_C - High P/E ratio diet with carbohydrate as main non-protein energy source; L_P/E_F - Low P/E ratio diet with fat as main non-protein energy source; L_P/E_C - Low P/E ratio diet with carbohydrate as main non-protein energy source.

### Nitrogen, fat and energy balance

Table 4 presents the apparent nutrient and energy digestibility coefficients (ADC) used to calculate parameters of nitrogen, fat and energy balance presented in Table 5. Digestible nutrient intakes in terms of digestible nitrogen intake (DNI), digestible fat intake (DFI) and DEI were different between the dietary groups. DNI was affected (P<0.001) by P/E ratio and the source of NPE without interaction between both factors (P>0.3). The DNI was 54% higher with H_P/E_ than L_P/E_ diets and 18% lower in diets with carbohydrate compared to fat as NPE source. Despite the differences in DNI between both H_P/E_ diets, RN was similar in trout fed the H_P/E_F and H_P/E_C diets. However, with L_P/E_ diets, retained nitrogen (RN) differed significantly in line with their DNI. DFI was affected (P<0.05) by the interaction between P/E ratio and NPE source of diet, being the lowest and the highest respectively in H_P/E_C and L_P/E_F diets. In contrast to DFI, retained fat (RF) was only influenced by the dietary NPE source, with 46% higher RF in trout fed fat relative to carbohydrate diets.

**Table 4:** Apparent nutrient digestibility coefficient (%; ADC) in rainbow trout fed with four experimental diets^1^.

	Diets^2^					*P*- value		
*Unit in %*	H_P/E_F	H_P/E_C	L_P/E_F	L_P/E_C	Pooled SEM	P/E ratio	NPE source	P/E×NPE
Dry matter (DM)	72.7^a^	83.8^b^	73.7^a^	80.1^b^	0.88	0.156	<0.001	0.027
Protein	95.5	95.9	96.1	95.2	0.24	0.750	0.338	0.028
Fat	96.7^a^	89.0^b^	95.8^a^	96.7^a^	0.38	<0.001	<0.001	<0.001
Total carbohydrates^3^	23.0^a^	76.4^b^	56.3^c^	74.0^b^	2.03	<0.001	<0.001	<0.001
Ash	34.0	36.5	32.7	33.8	1.99	0.346	0.396	0.709
Energy^3^	82.0^a^	88.7^b^	82.1^a^	85.7^c^	0.64	0.053	<0.001	0.040

1 Values represent least squares (LS) means (n = 3), row means with different superscript letters were significantly different and assigned only if interaction effect was significant (P<0·05).

2 H_P/E_F - High P/E ratio diet with fat as main non-protein energy source; H_P/E_C - High P/E ratio diet with carbohydrate as main non-protein energy source; L_P/E_F - Low P/E ratio diet with fat as main non-protein energy source; L_P/E_C - Low P/E ratio diet with carbohydrate as main non-protein energy source.

3 ADC of total carbohydrates and energy includes the effect of the added cellulose (indigestible) in diets H_P/E_F and L_P/E_F.

**Table 5:** Nitrogen, fat and energy balance in rainbow trout fed the experimental diets for 6 weeks^1^.

	Diets^2^					*P*- value		
	H_P/E_F	H_P/E_C	L_P/E_F	L_P/E_C	Pooled SEM	P/E ratio	NPE source	P/E×NPE
*Nitrogen balance (mg N kg^−0.8^ d^−1^)*								
GNI	1384	1204	680	484	15.1	<0.001	<0.001	0.593
FN	62.1	49.2	26.7	23.3	2.6	<0.001	0.011	0.103
DNI	1240	1068	620	437	13.7	<0.001	<0.001	0.393
BUN	905^a^	748^b^	367^c^	304^c^	16.3	<0.001	<0.001	0.021
RN	417^a^	408^a^	287^b^	157^c^	12.9	<0.001	<0.001	0.002
*Fat balance (mg kg^−0.8^ d^−1^)*								
GFI	2532^a^	501^b^	3198^c^	1661^d^	55.8	<0.001	<0.001	0.002
FF	84^a^	55^a^	136^b^	55^a^	7.2	0.005	<0.001	0.004
DFI	2448^a^	446^b^	3062^c^	1606^d^	56.8	<0.001	<0.001	0.001
RF	2011	1133	2093	1072	101	0.919	<0.001	0.496
RF/DF	0.83	2.54	0.68	0.67	-	-	-	-
*Energy balance (kJ kg^−0.8^ d^−1^)*								
GEI	380	303	352	246	6.6	<0.001	<0.001	0.055
FE	68	34	63	35	1.9	0.278	<0.001	0.133
DEI	311^a^	269^b^	289^ab^	211^c^	6.7	<0.001	<0.001	0.027
BUE	22^a^	19^b^	9^c^	7^c^	0.4	<0.001	<0.001	0.021
MEI	288^a^	250^b^	280^a^	203^c^	6.5	0.003	<0.001	0.018
RE	144	107	131	70	6.1	0.003	<0.001	0.083

SEM, Standard error mean; GNI, Gross nitrogen intake; FN, Faecal nitrogen loss; DNI, Digestible nitrogen intake; BUN, Branchial and urinary nitrogen loss; RN, Retained nitrogen; GFI, Gross fat intake; FF, Faecal fat loss; DFI, Digestible fat intake; RF, retained fat; RF/DF, fat efficiency; GEI, Gross energy intake; FE, faecal energy loss; DEI, digestible energy intake; BUE, branchial and urinary energy loss; MEI, metabolisable energy intake; RE, retained energy.

1 Values represent least squares (LS) means (n = 3), row means with different superscript letters were significantly different and assigned only if interaction effect was significant (P<0·05).

2 H_P/E_F - High P/E ratio diet with fat as main non-protein energy source; H_P/E_C - High P/E ratio diet with carbohydrate as main non-protein energy source; L_P/E_F - Low P/E ratio diet with fat as main non-protein energy source; L_P/E_C - Low P/E ratio diet with carbohydrate as main non-protein energy source.

**Figure 1:**
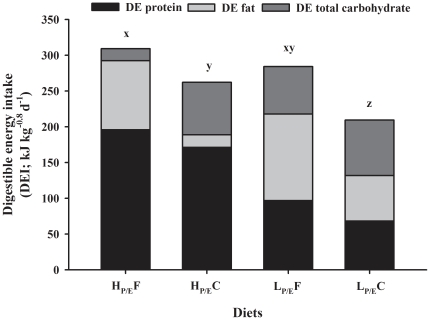
Effect of diet composition on digestible energy intake (DEI) in rainbow trout. Fish were fed to satiation with iso-energetic diets of different macronutrient composition having contrast in P/E ratio (high, H_P/E_ vs. low, L_P/E_) and NPE source (fat, F vs. carbohydrates, C) for 6 weeks. The bars show the amount of DEI derived from the digestible protein, fat and total carbohydrate (nitrogen-free extract) for each dietary group. Different superscripts indicate significant differences in total DEI.

**Figure 2:**
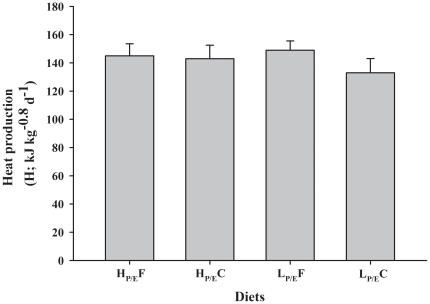
Effect of diet composition on heat production in rainbow trout. Heat production (H; least squares mean ± SD) in rainbow trout fed to satiation the iso-energetic diets of different macronutrient composition having contrast in P/E ratio (high, H_P/E_ vs. low, L_P/E_) and NPE source (fat, F vs. carbohydrates, C). H was unaffected by P/E ratio, NPE source and their interaction effect (P>0.05).

The amount of voluntary DEI, as supplied from the different dietary macronutrients, is shown in Fig. 1. The DEI paralleled dry matter intake, showing a significant interaction between dietary P/E ratio and NPE source (Table 5). The lowest DEI were observed in L_P/E_C fed groups, whereas DEI of trout fed diet L_P/E_F were not significantly different from those in H_P/E_ groups. There was no significant difference in metabolisable energy intake (MEI) between H_P/E_F and L_P/E_F groups, both being higher than in groups fed carbohydrate as NPE source. However, retained energy (RE) was different and significantly affected by both P/E ratio and NPE source of diet, being lower in trout fed L_P/E_- relative to H_P/E_-diets and in trout fed carbohydrate relative to fat as NPE source. Although DEI and RE was different, the total heat production (H) was unaffected (P>0.05) by the P/E ratio, the NPE source and their interaction (Fig. 2).

### Post-prandial glucose and triglyceride circulating levels

**Figure 3:**
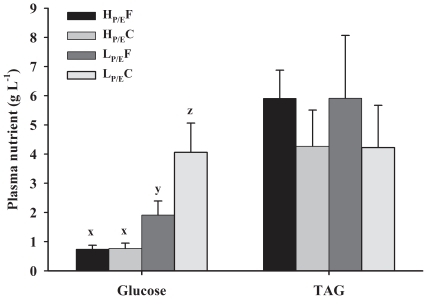
Effect of diet composition on post-prandial plasma glucose and triglycerides in rainbow trout. Seven hours post-prandial plasma levels (least squares mean ± SD) of glucose and triglycerides (TAG) of rainbow trout fed diets having contrast in P/E ratio and NPE source. Glucose was affected by dietary P/E ratio, NPE source and their interaction (P<0.001). In contrast, TAG levels were affected only by the NPE source (P = 0.003) and not by P/E ratio and their interaction effect (P>0.05).

Figure 3 depicts the 7 h post-prandial plasma glucose and triglyceride (TAG) levels in rainbow trout fed the four experimental diets. The plasma glucose (g L^−1^) was affected (P<0.001) by the dietary P/E ratio, NPE source and their interaction. Plasma glucose being higher in trout fed the L_P/E_ compared to H_P/E_ diets. The effect of NPE source on plasma glucose was significantly greater with the L_P/E_ diets than H_P/E_ diets. H_P/E_F and H_P/E_C diet showed similar plasma glucose levels and fish fed L_P/E_C diet attained the highest glucose levels. In contrast, TAG levels were affected by the NPE source (P = 0.037), being higher in trout fed fat vs. carbohydrate, but not (P>0.05) by the P/E ratio. There was no interaction between P/E ratio and NPE source on plasma TAG.

## Discussion

In the present study, voluntary FI paralleled DEI due to the similar DE contents of the formulated diets. FI in rainbow trout as in several other fish species has been reported to be regulated by the total DE content of the diet [3], [4]. The present data show that under satiation feeding conditions, rainbow trout consumed different amounts of DE, depending on the diet composition. These findings agree with previous reports in rainbow trout [11], [12], [22], highlighting the controversy on whether FI is adjusted to maintain a constant DEI in fish. In addition, these findings further suggest the involvement of dietary or physiological factors other than dietary DE content alone in the regulation of FI.

Independent of dietary DE level, FI has been shown to be directed by the animal's genetic growth potential in such a way that the animal will attempt to eat as much of a feed as needed to fulfil the nutrient requirements for achieving its (maximal) growth potential [23]. In this respect, intakes of specific nutrients such as protein have been shown to be separately regulated from energy intake, as shown in pig [24], poultry [25] and rat [26]. As a result, an excess of energy is ingested with low protein diets while an energy deficit may occur with high protein diets. Also fish have been reported to show hyperphagia and over-consume DE to compensate for reduced dietary protein as seen in Atlantic salmon [13]. In contrast, protein levels above optimum do not seem to down-regulate DEI in rainbow trout [11] in line with findings in mammalian carnivores used to deal with high protein intakes [27], [28]. The present low P/E (L_P/E_) and high P/E (H_P/E_) diets provided respectively 14 and 26 mg of digestible protein per kJ DE being, respectively, above and below the optimal DP/DE ratio of 17–19 mg kJ^−1^ [29] or 21 mg kJ^−1^ [30] for rainbow trout. However, the similar or even decreased DEI in L_P/E_- compared with the H_P/E_-groups show that the trout fed the low P/E diets did not ‘over-eat’ energy to compensate for the reduced protein. In both cases, this resulted in lower digestible nitrogen intake (DNI) as well as lower weight and protein (RN) gain than with the high P/E diets.

According to the lipostatic theory of FI regulation [31], the failure of the trout fed L_P/E_ diets to increase DEI and hence compensate DNI may be caused by the higher relative level of body fatness of fish fed the L_P/E_ compared with H_P/E_ diets. The negative effect of high body fat content on FI or DEI [31], mediated through the feedback mechanism of leptin is well documented in mammals [32]. Adipostatic feedback control of FI has also been reported to occur in salmonid fish [29], [33], [34]. However, diet-induced increases in the relative level of adiposity, which moreover varies depending on body size [35], did not necessarily reduce appetite or energy intakes in rainbow trout [11], [36]. Similarly, the observation of similar DEI in trout fed H_P/E_C and L_P/E_F diets, despite the difference in adiposity (61 and 111 g kg^−1^, respectively), suggests a low feedback control of relative body fatness on DEI.

Interestingly, rainbow trout reduced intakes following the iso-energetic substitution of fat by carbohydrate, irrespective of the dietary P/E ratio. This might be due to physical constraints as the volume of feed a fish can eat depends on the stomach capacity and gut evacuation rate [37], [38]. The expansion of starch during feed extrusion reduces the bulk density of the pellets. As such, the lower density of diet L_P/E_C possibly limited the amount of FI during the first meals, but unlikely affected the long term (weeks) FI, as fish are known to increase stomach volume when fed high-bulk diets [39]. In addition, gut evacuation rate and hence the return of appetite are expected to be enhanced by the relatively high (16°C) water temperature [40]. Another factor susceptible to reduce FI following the substitution of fat by carbohydrate is increased plasma glucose. The glucostatic theory implies that FI is controlled to maintain glucose homeostasis in blood through a feedback mechanism signaled by both hypothalamus and liver [41]. Thus, an increase or decrease in blood glucose level leads respectively to a down- or up-regulation of FI. Evidence in fish on glucostatic control of FI is highly ambiguous. For instance, high plasma glucose was found to either increase [42] or decrease [43], [44] FI in fish. Our data on the relation between FI and plasma glucose also appear inconsistent as the substitution of fat by carbohydrate either increased (L_P/E_-groups) or unmodified (H_P/E_-groups) plasma glucose, whereas this led to reduced intakes in both groups. Moreover, voluntary FI between H_P/E_C and L_P/E_F groups were not significantly different, despite the differences in circulating plasma glucose.

Rather than a direct glucostatic or lipostatic feedback control of FI, some studies in mammals suggest that it is the overall metabolic utilization of the ingested nutrients which signals satiety and hence determines FI [45], [46], [47]. In other words, the degree of nutrient oxidation rather than the ingested amount of dietary energy *per se* would generate satiety [48]. In fish, the question whether and how dietary energy utilization (energy retention vs. expenditure/heat production) regulates the amount of DEI has received little attention. Interestingly, the energy balance of the present trout revealed no significant difference in heat production (133–149 kJ kg^−0.8^ d^−1^) between fish of the different treatments, whereas the amount of energy retained (70–144 kJ kg^−0.8^ d^−1^) and DEI (211–311 kJ kg^−0.8^ d^−1^) were strongly affected by the dietary DE source. This confirms previous findings in Nile tilapia fed to satiation with diets varying in macronutrient supply and supports the hypothesis that heat production may set a limit to voluntary FI [16]. This was also suggested in the very early works of Brobeck [49] in mammalian models, reporting that the important factor in FI regulation is not the food's energy value, but rather the amount of extra heat released during its assimilation. Further studies with homoeothermic vertebrates confirmed the relation between heat production and FI, yet mostly in relation with ambient temperature [50]. Homoeothermic animals, when exposed to ambient temperature above the upper critical temperature, lower FI in order to avoid the excess heat production caused by the thermic effect of feeding [50]. As such, the extent to which the animal is able to dissipate heat to the environment will determine how much it will eat, as shown in pig [51] and broiler [52]. Since fish do not maintain constant body temperature, the amount of heat to be dissipated to the environment is not expected to control FI in fish in the same way as in homeotherms. Therefore, other more basic metabolic processes involved in heat production, shared by both homeo- and ectotherms, such as aspects related with oxygen use, may be implicated in the dietary control of FI in fish.

**Figure 4:**
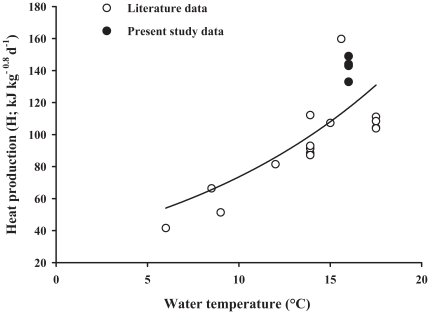
Relation between water temperature (T, °C) and heat production in rainbow trout fed to satiation. The heat production values (H, kJ kg^−0.8^ d^−1^) are calculated for rainbow trout fed to satiation from literature data [57], [58], [59], [60], [61] and from the present study. H was curvilinearly related to temperature, H = 26.6×e^0.0923×T^, R^2^ = 0.73.

Theoretically, the amount of heat production by aerobic metabolism in animals parallels the amount of oxygen consumed [53]. In mammals, several studies pointed at the difference between macronutrients in their contribution to oxidative metabolism and how these may relate to satiety [46], [48]. In this respect, satiety and hence dietary FI control have been associated with the degree of hepatic oxidative metabolism [54], [55] or the efficiency of oxygen use [56]. The comparison of the heat production values observed in the present study (133–149 kJ/kg^0.8^/d) with values calculated (i.e., H = MEI-RE) from literature for rainbow trout fed to satiation (e.g., 107 [57], 77–91 [58], 93–112 [59], 160 [60] and 103–112 [61] kJ/kg^0.8^/d), shows our values to be in the upper range, even after adjusting for the effect of temperature (positive curvilinear relationship between both variables, Figure 4). The present finding that heat production was similar irrespective of dietary composition in trout kept under normoxic condition, suggests that the DEI control in fish is a function of heat production. This might reflect a physiological limit related to oxidative metabolism. Various biological constrains might cause such a limit in fish even under normoxic water condition. For instance, the capacity of oxygen uptake by the fish (e.g. gill surface [16]), the capacity of oxygen transport (e.g. cardiac performance, hemoglobin affinity for O_2_) and/or constraints in oxidative metabolism at cellular level (e.g. mitochondrial respiration, production of reactive oxygen species). Measurements of oxygen consumption data are needed to further elucidate the role and possible limits set by heat production/oxidative metabolism on DEI. Therefore, ongoing studies in our laboratories further explore the relation between macronutrient-induced changes in feed/nutrient intake and oxygen consumption as well as the link with hepatic oxidative metabolism and hypothalamic satiety markers.

In conclusion, the present study demonstrates that the macronutrient composition of the diet modifies voluntary DEI in rainbow trout. The observation that the rainbow trout had similar heat production, together with different DEI, is in line with the proposed hypothesis that DEI in fish might be controlled as a function of heat production, which might reflect a physiological limit related to oxidative metabolism.
